# Supplementary figures and images for: Comparative test-retest variability of outcome parameters derived from brain [18F]FDG PET studies in non-human primates
Source: PLoS One. 2020 Oct 5;15(10):e0240228. doi: 10.1371/journal.pone.0240228 (PMC7535063; doi:10.1371/journal.pone.0240228)

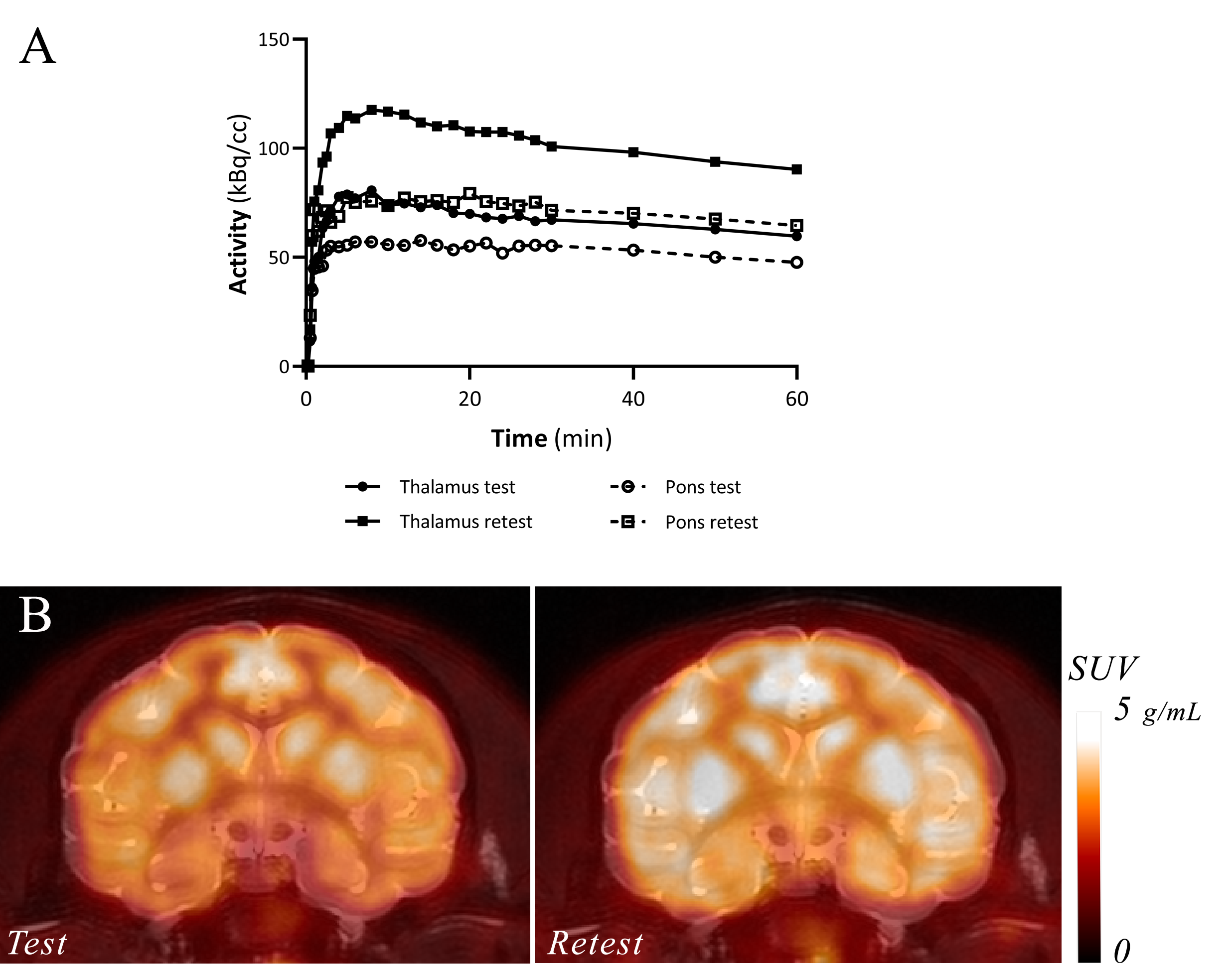

Supplement: S1 Fig — A)- Time activity curves of a representative animal in the pons and thalamus regions; B)- SUV PET images of the same animal at test and retest sessions. (TIFF) [file pone.0240228.s001.tiff]
